# The Effect of Isotonic Saline Nasal Lavages in Improving Symptoms in SARS-CoV-2 Infection: A Case-Control Study

**DOI:** 10.3389/fneur.2021.794471

**Published:** 2021-12-06

**Authors:** Giacomo Spinato, Cristoforo Fabbris, Giulio Costantini, Federica Conte, Pier Giorgio Scotton, Francesco Cinetto, Rosalba De Siati, Alessandro Matarazzo, Marco Citterio, Giacomo Contro, Cosimo De Filippis, Carlo Agostini, Enzo Emanuelli, Paolo Boscolo-Rizzo, Daniele Frezza

**Affiliations:** ^1^Department of Neurosciences, University of Padua, Padua, Italy; ^2^Department of Otolaryngology, Ospedale di Treviso, Treviso, Italy; ^3^Department of Psychology, University of Milano-Bicocca, Milan, Italy; ^4^Department of Otolaryngology, University of Trieste, Trieste, Italy

**Keywords:** SARS-CoV-2 infection, COVID-19, nasal lavage, upper airways infection, nasal swab

## Abstract

**Background:** Severe Acute Respiratory Syndrome Coronavirus 2 (SARS-CoV-2) mainly colonizes nasopharynx. In upper airways acute infections, e.g., the common cold, saline nasal irrigations have a significant efficacy in reducing symptoms. The present study aimed to test the efficacy of nasal lavages in upper airways symptoms of Coronavirus Disease 2019 (COVID-19).

**Methods:** A series of consecutive adult subjects who tested positive for SARS-CoV-2 from December 2020 to February 2021 performed daily nasal lavages with saline solution (Lavonase®—Purling, Lugo di Romagna, Italy) for 12 days, starting on the day after the SARS-CoV-2 positive swab. A control group included a historical series of patients who were infected in February-March 2020 and who did not perform lavages. An *ad hoc* questionnaire regarding symptoms was administered to each subjects at base-line and 10 days after diagnosis (i.e., on the same day of the control swab) in both cases and controls.

**Results:** A total of 140 subjects were enrolled. 68 participants in the treatment group and 72 in the control group were included. 90% of respondents declared the lavages were simple to use and 70% declared they were satisfied. Symptoms of blocked nose, runny nose, or sneezing decreased by an average of 24.7% after the treatment. Blocked nose and sneezing increased in the same period of time in the control group. Ears and eyes symptoms, anosmia/ageusia symptoms, and infection duration (10.53 days in the treatment group and 10.48 days in the control group) didn't vary significantly among the two groups.

**Conclusion:** Nasal lavages resulted to significantly decrease nasal symptoms in newly diagnosed SARS-CoV-2 patients. These devices proved to be well-tolerated and easy to be used. Further studies on a larger number of subjects are needed in order to possibly confirm these preliminary results.

## Introduction

During the last year, the current pandemic situation has brought clinicians to an ongoing quest toward the identification of novel tools to manage Severe Acute Respiratory Syndrome Coronavirus 2 (SARS-CoV-2) infection. In particular, the management of asymptomatic or oligosymptomatic patients represents a challenge, also in terms of development of prophylactic strategies to prevent the manifestation or worsening of clinically relevant symptoms, as well as to reduce the viral transmission (1, 2).

As in most of the respiratory infections, including influenza, also in SARS-CoV-2 viral shedding reaches the highest level in the nasopharynx, being also nasal cavity mucosa as one of the most relevant sites of viral activity (3–5). In previous studies on other respiratory infections, including common cold, saline nasal irrigations have been applied as a topical treatment approach, showing a significant efficacy in reducing symptom burden and decreasing viral shedding (6). This observation has led clinicians to focus their interest on the feasibility of a topical management of SARS-CoV-2 infection, based on the reduction of viral load in the nasal cavities and into the upper airways. However, although some trials are currently ongoing (7, 8), to date few and sparse evidence supporting topical preventive or therapeutic strategies in managing SARS-CoV-2 infection are available in the literature (9, 10).

Recently, three Cochrane reviews explored the evidence supporting the use of antimicrobial mouthwashes and nasal spray as a preventive tool to protect healthcare workers when performing aerosol-generating procedures (11) and when assisting suspected or confirmed Coronavirus Disease 2019 (COVID-19) cases (12), or as a therapeutic strategy to improve the outcome of patients with SARS-CoV-2 infection (13). However, none of these meta-analyses found in the literature provided sufficient evidence to support such strategies. Moreover, the large majority of the available reports on topical treatment of SARS-CoV-2 infection regards the local administration of antimicrobial solutions. On the other hand, the preventive and therapeutic role of isotonic saline solution nasal lavages has yet to be extensively explored.

The rationale of proposing isotonic saline solution lavages in SARS-CoV-2 infection resides not only in the mechanical action of the injected fluid which clears the viral particles out of the nasal fossae (14), but also, as recently reported (15), in a direct anti-microbial effect of saline solution, which may allow the epithelial cells to produce hypochlorous acid (HOCl).

Based on this rationale, and given the potential therapeutic relevance of this practice the principle aim of the present investigation was to evaluate the effectiveness of isotonic saline nasal lavages in improving symptoms of COVID-19. Secondary aims were to verify whether nasal lavages may reduce the incidence of symptoms in patients with asymptomatic SARS-CoV-2 infection and to evaluate the compliance to the use of a nasal-lavage device.

## Materials and Methods

### Study Design

The present study was approved by the ethics committee of Treviso and Belluno provinces (ethic vote: 871/CESC). All patients included in this study received specific information material and signed a detailed informed consent form.

This study was a non-randomized controlled trial. The treatment group included a series of consecutive patients which underwent nasal lavages (see also paragraph “***Treatment***”), while the control group included an historical cohort, matched for age, sex, and base-line symptoms.

The date of the first negative test was also collected for each patient.

### Treatment Group

A series of consecutive subjects who received diagnosis of SARS-CoV-2 infection in a period from December 9th 2020 to February 25th 2021 were included in the treatment group.

Inclusion criteria were:

1. positive molecular test for SARS-CoV-2 infection,
2. age ≥18 years,
3. capability of self-performing nasal lavages.

Exclusion criteria were:

1. clinical conditions preventing self-administration of nasal lavages,
2. clinical conditions preventing administration of the symptom questionnaire,
3. refusal to take part in the study.

### Treatment

Nasal lavages were self-performed by each patient in the treatment group by the mean of a device, Lavonase® (Purling, Lugo di Romagna, Italy), which injected the saline solution into a nasal fossa, allowing it to enter the nasopharynx and to be evacuated from the other nasal fossa. Each nasal lavage administrated 250 ml of saline isotonic solution (NaCl 0.9%). The treatment schedule included one daily nasal lavage for 12 days, starting on the day after the molecular diagnosis of SARS-CoV-2 infection.

### Symptom Questionnaire

The COVID-Q questionnaire on SARS-CoV-2 infection symptoms (16) was administered to each patient, at base-line and 10 days after diagnosis. The questionnaire included questions on the main clinical presentation patterns of SARS-CoV-2 infection: asthenia, influenza-like symptoms, ear and nose symptoms, breathing issues, throat symptoms, and altered sense of smell or taste (16). From those data, symptoms regarding the otolaryngologic field were considered and analyzed. Among questions about patients' history, one regarding “other not previously specified” was clearly asked, including sinonasal diseases.

When repeated 10 days after diagnosis, two further questions were added, regarding the ease of use of the device and the subjective satisfaction after treatment.

### Control Group

The control study included a historical series of patients who tested positive for SARS-CoV-2 in a period from February 19th to March 23rd 2020 and who answered the COVID-Q questionnaire on the following day and on the 10th day since diagnosis.

This series was statistically comparable with the treatment group according to age, sex, and base-line symptoms.

Patients in both treatment and control group underwent a control molecular test for SARS-CoV-2 10 days after diagnosis. If they still tested positive at day 10, they would receive another test 7 days later.

In line with other studies in the field, the sample size was estimated according to a sensitivity analysis, which showed that 70 subjects provided 80% power to detect an effect size as low as dz = 0.307 in a one-tailed Wilcoxon signed-rank test, at the conventional alpha level of 0.05. One-hundred-forty subjects provided 80% power to detect an effect size as small as *d* = 0.42 in a one-tailed *t*-test, at the conventional alpha level of 0.05.

### Statistical Analysis

The aim of the study was to examine the impact of nasal lavages on COVID-19, with regards to symptom frequency. First, participants' experiences with the intervention were assessed, testing for age and sex effects. Given the ordinal scale of the compliance variables, sex differences were investigated through the non-parametric Mann-Whitney U test, and age effects through Spearman's rank-order correlations.

COVID-19 symptoms have been shown to follow different trajectories during the infection. Therefore, each symptom was analyzed individually. Baseline symptoms were compared between the treatment and the control group. Next, change in symptom frequency across occasions was analyzed for each group separately using Wilcoxon's signed-rank test. The results also report the proportion of participants experiencing symptoms in the two groups.

Finally, an independent sample *t*-test was used compare the duration of the infection in the case and in the control group.

## Results

140 Subjects Were Enrolled and Divided Into two Groups. The Treatment Group Included 68 Participants (35 Males and 33 Females; Mean age 49.2 Years, Range 18–75 Years). The Control Group Comprehended 72 Subjects (29 Males and 43 Females; Mean age 49.2 Years, Range 21–75 Years). As Intended, There were no Significant Differences in the Mean age or sex Composition of the two Groups. In the Overall Sample, Women Were on Average 4.6 Years Younger than men: *t* (209) = −2.21, *p* = 0.028. The Mean age was 46.7 Years in Women and 51.3 in men.

Participants in the treatment group were asked to report on ease of use and satisfaction with the treatment. Sixty out of 68 participants answered the questions. The lavages appeared simple to use, with 90% (*N* = 54) of respondents marking them as “easy” or “extremely easy”. Furthermore, the answers indicated a good satisfaction with the treatment, with 70% (*N* = 42) of participants declaring themselves “satisfied”, “very satisfied” or saying they “would suggest [the lavages] to others”. Mann-Whitney U test showed that the experience did not vary significantly according to sex (*W* = 471.5, *p* = 0.709 for ease of use, *W* = 553.5, *p* = 0.113 for satisfaction), nor did it correlate significantly with age (*r* = 0.11, *p* = 0.376 for ease of use, *r* = 0.10, *p* = 0.437 for satisfaction).

None of the patients reported sinonasal diseases or others possibly having an influence on nasal function, previous to infection. Table 1 reports symptom frequency at the first and second assessment for the treatment and control group. Group differences in baseline symptoms were analyzed using Mann-Whitney U test (Table 2).

**Table 1:** Reported symptom frequency.

	**First assessment**							**Second assessment**						
	**0**	**1**	**2**	**3**	**4**	**5**	**prop symptom**	**0**	**1**	**2**	**3**	**4**	**5**	**prop symptom**
**Intervention**														
Painful pressure in ears^1^	51	16	1	–	–	–	0.25	57	11	0	–	–	–	0.16
Blocked nose^1^	31	32	5	–	–	–	0.54	52	15	1	–	–	–	0.24
Runny nose^1^	40	26	2	–	–	–	0.41	58	9	1	–	–	–	0.15
Sneezing^1^	49	18	1	–	–	–	0.28	61	7	0	–	–	–	0.10
Watery eyes^1^	61	7	0	–	–	–	0.10	65	3	0	–	–	–	0.04
Altered sense of smell or taste^2^	33	8	7	5	6	9	0.73	41	6	7	4	3	7	0.50
**Control**														
Painful pressure in ears^1^	66	6	0	–	–	–	0.08	61	8	3	–	–	–	0.15
Blocked nose^1^	63	7	2	–	–	–	0.13	38	28	6	–	–	–	0.47
Runny nose^1^	56	16	0	–	–	–	0.22	52	16	4	–	–	–	0.28
Sneezing^1^	58	14	0	–	–	–	0.19	46	22	4	–	–	–	0.36
Watery eyes^1^	66	6	0	–	–	–	0.08	60	11	1	–	–	–	0.17
Altered sense of smell or taste^2^	56	0	1	1	2	12	0.28	61	0	0	3	2	6	0.18

*Note. Prop symptom, proportion of patients scoring 1 or higher on the symptom frequency over total number of patients in the group*.

1 *Symptom Frequency Assessed on a 0−2 Scale, 0 = not Experienced, 1 = Experienced a Little, 2 = Experienced a lot*.

2 *Symptom Frequency Assessed on a 0−5 Scale, 0 = not Experienced, 1 = Experienced Barely, 2 = Experienced a Little, 3 = Experienced Moderately, 4 = Experiences a lot, 5 = Complete Loss of Smell or Taste*.

**Table 2:** Test of between-group differences in symptom frequency at first assessment.

	**U**	**p-value**
Painful pressure in ear	2,859	0.008
Blocked nose	3,459.5	0.000
Runny nose	2,928	0.013
Sneezing	2,663	0.224
Watery eyes	2,496	0.694
Altered sense of smell or taste	3,003	0.007

*Note. U, U statistic from Mann-Whitney U test*.

**Figure 1:**
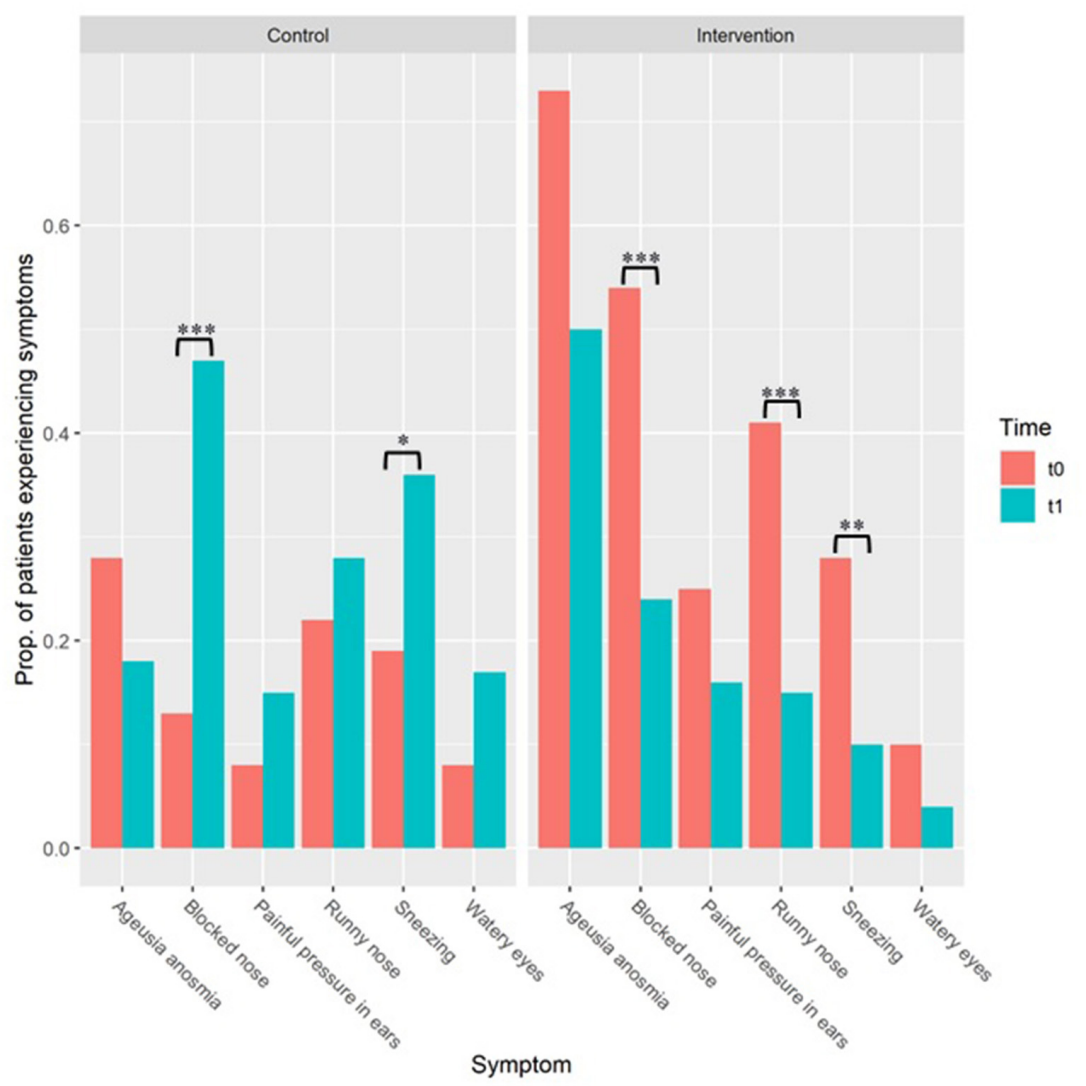
Proportion (“Prop.” within the figure) of patients reporting otolaryngologic COVID-19 symptoms. “t0” refers to baseline. “t1” refers to follow-up period after 10 days. Results of the control group are reported on the left (“Control”) and those of the treatment group are reported on the right (“Treatment”). Note. *p ≤ 0.05, **p ≤ 0.01, ***p ≤ 0.001.

The change in symptoms across time was investigated within each group separately. Table 3 reports statistics and significance levels from Wilcoxon's signed-rank test and Figure 1 illustrates the proportion of patients experiencing symptoms. The frequency of the blocked nose and sneezing symptoms varied significantly in both the treatment and the control group. In the treatment group, the proportion of participants experiencing a blocked nose, either occasionally or frequently, decreased by 30.9% (i.e., from 54.4 to 23.5%), and patients experiencing sneezing decreased by 17.6% (i.e., from 27.9 to 10.3%). The control group showed the opposite trend, as the number of people reporting symptoms increased significantly across occasion: 24.2% more patients reported a blocked nose and 16.7% more patients reported sneezing (i.e., increasing from 13.2 to 37.4% and from 19.4 to 36.1%, respectively) (Table 1).

**Table 3:** Test of within-group differences in symptom frequency between assessments.

	**Intervention**		**Control**	
	**V**	***p*-value**	**V**	***p*-value**
Painful pressure in ear	60	0.078	32.5	0.103
Blocked nose	510	0.000	39	0.000
Runny nose	264	0.000	203	0.217
Sneezing	198	0.010	170.5	0.018
Watery eyes	27	0.182	40	0.115
Altered sense of smell or taste	442.5	0.084	175.5	0.247

*Note. V, V statistic from Wilcoxon's signed-rank test*.

The runny nose symptom showed a significant decline (i.e., from 41.2 to 15.1% of participants) with the treatment, and no significant change in the control group. The painful pressure in ears, watery eyes, and anosmia/ageusia symptoms did not vary significantly across occasions in either the case or the control group (Table 1, Figure 1).

The infection lasted on average 10.53 days (range = 7–26, sd = 3.5) in the treatment group and 10.48 days (range = 6–31, sd = 3.95) in the control group. The *t*-test for independent samples confirmed that there was no significant difference between the mean infection duration in the two groups (*t* (137) = 0.08, C.I. = −1.202; 1.304, *p* = 0.936).

Follow-up molecular test at 10 days resulted negative among 62 cases (91.1 %) and in 2 controls (2.8%), with a statistically significant difference (*p* < 0.00001).

## Discussion

The present work was a pilot study investigating the effect of nasal lavages on COVID-19 symptoms. Our analysis showed that nasal lavages can significantly reduce the frequency of nose-related symptoms. Specifically, the proportion of patients experiencing a blocked nose, runny nose, or sneezing decreased by an average of 24.7% after the treatment. Conversely, over the same period of time, blocked nose and sneezing became more frequent in patients who did not perform the lavages. Thus, our results suggest that the treatment can offer a substantial relief from COVID-19-symptoms affecting the nose.

On the other hand, our study did not identify a significant difference in the evolution of non-nasal symptoms over time between patients who performed nasal lavages and those who did not. This seems to be in line with available evidence on other upper respiratory tract infections, not related with SARS-CoV-2, stating that nasal saline irrigation may be beneficial for nasal symptoms but not respiratory symptoms (17).

It is worth noting that, in our study population, although nasal symptoms seemed to worsen over time in the absence of treatment, they significantly improved in patients who performed nasal irrigation. This can be interpreted in view of both the well-known efficacy of saline nasal irrigations on symptoms of chronic sino-nasal inflammatory conditions and a possible direct effect in reducing the local viral load into the upper airways.

Literature reports that saline irrigation may improve the patient-reported severity of allergic rhinitis symptoms compared with no saline treatment in children and adults, both on the short-term (up to 4 weeks) and on the medium-term (4 weeks to 6 months) (18). Similar data emerged also from reports on non-allergic chronic sino-nasal inflammatory conditions (19). The effectiveness of nasal irrigation on chronic inflammatory sino-nasal symptoms has been described for isotonic (20), hypertonic (21), and mineral-enriched saline (22) solutions.

Regarding the effect of nasal irrigations on controlling the pathogen load in sino-nasal cavities, evidences seem to support the idea that saline solution alone may be as beneficial as direct antimicrobial agents (23), probably due to a possible direct antimicrobial effect of the hypochlorous acid, produced by the epithelial cells based on sodium chloride (15).

Other previously published papers studied the effectiveness of antimicrobial solutions (e.g., Amphotericin B) on sinonasal diseases (24, 25). Accordingly, no relevant reduction of chronic rhinosinusitis symptoms were obtained. Moreover, by comparing antimicrobial and saline solution, effects were not statistically different. Based on these results, antimicrobial properties of nasal irrigation seems not to be essential, thus confirming suitability of the saline solution we used in this study.

Our findings confirm previous literature with regards to the evolution of individual symptoms (26–28). Indeed, we observed that the frequency of anosmia and ageusia (i.e., loss of smell and taste), painful pressure in the ears, and watery eyes did not change significantly across measurement occasions, neither in the treatment nor in the control group. On the other hand, blocked nose and sneezing symptoms showed a greater, significant change in the observed time-span.

The nasal lavage treatment did not appear to affect the duration of the infection, as the range and mean infection duration did not differ significantly between the treatment and the control group. A statistically significant difference was obtained by comparing the rates of negative swabs among cases and controls at 10-day follow-up, showing a clearly higher rate among subjects who performed nasal lavages. However, such a comparison may be weakened by the fact that microbiological data were available only at fixed times, whereas a daily test might have detected subtler differences between the two groups in time to negativization.

Another weakness of this study concerns the relatively limited number of cases considered. However, based on the preliminary sample size analysis, it was deemed suitable to address this study's primary endpoint. Also, the modalities of treatment administration prevented the possibility of blinding, which might potentially reduce biases in patient's reports on symptoms.

On the other hand, the main strengths of this investigation lie in its controlled design and in the homogeneity of the series of patients considered because: only new diagnoses of SARS-CoV-2 infection were considered; all treated patients received the material for nasal lavage within 24 h from the diagnosis; the control group was comparable regarding age, sex and symptoms at the baseline.

In conclusion, data from this preliminary study showed a good compliance and subjective satisfaction for nasal lavages in patients with newly diagnosed SARS-CoV-2 infection. The treatment showed effectiveness in reducing nasal symptoms of SARS-CoV-2 infection, compared to the control group. However, further studies on larger scale are advocated to better characterize the effectiveness of this treatment on non-nasal symptoms and on the time to microbiological remission.

## Data Availability Statement

The original contributions presented in the study are included in the article/supplementary material, further inquiries can be directed to the corresponding author/s.

## Ethics Statement

The studies involving human participants were reviewed and approved by Comitato Etico Ospedaliero di Treviso e Belluno. The patients/participants provided their written informed consent to participate in this study.

## Author Contributions

GS and CF: conceptualization, manuscript drafting, and manuscript supervision. GCos and FCo: data analysis and manuscript drafting. PS, FCi, RD, AM, MC, GCon, CD, and CA: data collection and manuscript drafting. EE, PB-R, and DF: data collection, manuscript drafting, and manuscript supervision. All authors contributed to the article and approved the submitted version.

## Conflict of Interest

The authors declare that the research was conducted in the absence of any commercial or financial relationships that could be construed as a potential conflict of interest.

## Publisher's Note

All claims expressed in this article are solely those of the authors and do not necessarily represent those of their affiliated organizations, or those of the publisher, the editors and the reviewers. Any product that may be evaluated in this article, or claim that may be made by its manufacturer, is not guaranteed or endorsed by the publisher.
